# Occurrence and identification of Emeria species in broiler rearing under traditional system

**DOI:** 10.1186/s40781-015-0074-0

**Published:** 2015-12-01

**Authors:** Asim Shamim, Murtaz ul Hassan, Arfan Yousaf, Muhammad Farooq Iqbal, Muhammad Arif Zafar, Rao Muhammad Siddique, Muhammad Abubakar

**Affiliations:** Faculty of Veterinary and Animal Sciences, The University of Poonch, Rawalakot, Azad Kashmir, Pakistan; Faculty of Veterinary Science, Arid Agriculture University, Rawalpindi, Pakistan; Department of Parasitology, Faculty of Veterinary Science, University of Agriculture, Faisalabad, Pakistan; National Veterinary Laboratory, Park Road, Islamabad, Pakistan

**Keywords:** Occurrence, Emeria species, Broilers, Azad Kashmir, Pakistan

## Abstract

**Background:**

The present study was intended to determine the prevalence and identification of species involved causing coccidiosis in broilers rearing under traditional farming system in Mirpur, Azad Kashmir, Pakistan. For the current study, a convenient sampling technique was carried out. A total of 7814 broilers (aged 1 to 6 weeks) were submitted to the Disease Diagnostic Laboratory of Livestock and Animal husbandry Department Mirpur, Azad Kashmir.

**Results:**

From the total screened, 750 were found positive for coccidiosis representing an overall prevalence of 9.59 %. Age-wise highest prevalence (10.88 %) recorded in the middle age birds (0 to 3 week old) were found more susceptible to infection than those aged above 3 weeks. Higher prevalence (12.49 %) of coccidiosis in broilers was observed in spring as compared with 6.60 % in summer season. In this study two main coccidiosis causing species, *Emeria tenella* and *Emeria maxima* were identified on the basis of their morphological feature and habitat (caeca and intestine), However, *E. tenella* was dominant compared to *E. maxima*.

**Conclusion:**

The study provides an insight to the occurrence of *Emeria* species which must be taken into consideration when rearing the broilers.

## Background

Coccidiosis, considered as a frequent and significant disease of poultry in general and in broiler specifically caused by the *Apicomplexan* protozoan parasites of the genus *Eimeria* (E) which lives and replicates in gut mucosa of broiler [1]. These are the main species that cause coccidiosis in broiler with varying degree of pathogenicity viz: *E. tenella, E. maxima, E. necatrix,* and *E. acervulina* dispersed globally [2].

Coccidiosis happened in two forms that are clinical, characterized by bloody diarrhoea, emaciation, drooping wings, stunted growth, pale comb, reduced production [3], with high mortality and morbidity rate [4], and subclinical it may render chickens immunocompromised thus providing opportunity for secondary infections [5]. It is considered as one of the expensive [6] and profit reducing disease as it impacts economy of production as reported in United States where it causes an annual loss of US$ 127 million [7].

The incidence of coccidiosis in marketable poultry (broiler) can range from 5 to 70 % [8], due to higher stocking densities [9] and improper managment practices [10] although much progress in medication and management has been employed. Furthermore, various factors such as daily shift in environmental temperature and the development of resistance against commonly available anticoccidial agents used for medication of coccidial infection contribute to higher prevalence of coccidiosis in poultry industry. Therefore, it is needed to plan the prevalence surveys to describe and quantify the disease burden in broilers in specific area. The present study was intended to determine the prevalence and identification of species involved causing coccidiosis in broilers rearing under traditional managmental farming system in district Mirpur, Azad Kashmir, Pakistan.

## Methods

### Study area

The present study was carried out at District Disease Diagnostic Laboratory Department of Livestock and Animal Husbandry Mirpur, Azad Kashmir that is situated at 33.11° and 33.34° latitude and 73.31° and 73.55° longitudes and 459 m above the sea levels. It has hot climate with an average maximum temperature of 40 °C. Topographically, district Mirpur consist of stretches of plains and small mud made peaks.

### Sampling methodology

The present study was conducted from January to December, 2011. For the current study, convenient sampling technique was applied. All the birds, which were submitted to the disease investigating laboratory (Department of livestock and animal husbandry) for diagnosis of different infections/diseases were examined. Moreover, all the mandatory information about numbers of birds reared, their age, dimensions of the farms, managmental condition, vaccination schedule, feed and medicine used, morbidity and mortality rate was noted on the pre-designed questionaire. All the procedures to conduct this research were carried out in the light of guidelines of National Chicken Council [11] for broilers.

### Parasitological procedures

#### Clinical and post mortem examination

The birds, which were directly brought to the laboratory and/or collected from the farms for diagnosis of diseases were observed through clinical and postmortem examination. For this purpose, entire gut of the collected broiler chickens was keenly observed for the manifestation of gross lesions and haemorrhages.

### Faecal examination

Gut contents were also examined through the direct smear method [12], for the presence and identification of oocysts of coccidian species based on their morphology and location in the gut [13]. The frequency of coccidiosis in broiler was recorded by microscopic examination.

### Statistical analysis

For the calculation of prevalence of coccidiosis in broiler data were analysed using statistical software package. Association among risk factors/variables associated with coccidiosis was determined by calculating odds ratio and P-values. It is considered statistically significant if the P-value less than 0.05 and confidence interval (CI) 95 %.

## Results

During January 2011 to December 2011, a total of 7814 broiler aged 1 to 6 weeks were submitted to the Disease Diagnostic Laboratory of Livestock and Animal husbandry Department Mirpur, Azad Kashmir. Among the total birds screened, 7064 were found negative for coccidiosis and 750 were found positive for coccidiosis representing an overall prevalence of 9.59 %. Two main coccidiosis causing species, *Emeria tenella* and *Emeria maxima* were identified on the basis of their morphological feature of oocyst of both species and site of habitat (caeca and intestine). However, *E. tenella* was dominant species compared to *E. maxima*. When broilers were divided into two age groups: 0–3 week old vs. 3–6 week old, chicks aged less than 3 weeks had higher prevalence (10.88 %) than those aged above 3 weeks. Monthwise higher prevalence was recorded in the month of March while lowest (2.46 %) prevalence was noted in the month of June (Table 1). Likewise, seasonal prevalence was also recorded which indicated that seasonwise higher prevalence (12.49 %) of coccidiosis in broiler was observed in spring followed by winter (9.03 %) autumn (8.73 %) and summer (6.60 %) in decreasing trend (Table 1). When the occurrence of coccidiosis was recorded on the basis of farm floor types, it was shown that higher (11.1 %) prevalence of coccidiosis was noted on farms with soiled floor compared with those with cemented floor (Table 1).

**Table 1:** Comparison of variables/risk factors associated with the occurrence of coccidiosis in broiler farms in district Mirpur, Azad Kashmir

Variables	Levels	Birds screened (*N*)	Positive (*n*)	Prevalence (%) (n/N × 100)	95 % C.I		Odds ratio (OR)	*P*-value
					Lower limit	Upper limit		
Age of Birds	0-3 Week	4109	447	10.88	56.05	63.07	1.33	0.000
	>3-6 Week	3705	303	8.18	36.93	43.95	-	-
Species	*E. tenella*	7814	421	5.39	52.56	59.66	1.28	0.001
	*E. maxima*	7814	329	4.21	40.34	47.44	-	-
Floor Pattern	Soiled	2935	323	11.01	39.55	46.63	1.62	0.000
	Semi Cemented	2645	275	10.40	33.27	40.16	1.53	0.000
	Cemented	2234	152	6.80	17.50	23.26	-	-
Seasonal Prevalence	Spring	2858	357	12.49	27.83	42.10	1.89	0.000
	Winter	1749	158	9.03	22.98	37.96	1.37	0.009
	Autumn	1100	96	8.73	30.54	44.32	1.32	0.042
	Summer	2107	139	6.60	51.29	63.63		

## Discussion

**Fig. 1:**
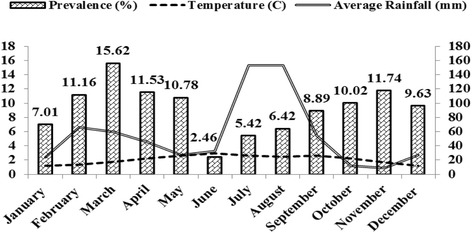
Month-wise prevalence along with environmental factor of coccidiosis in broiler

Study on reporting diseases have significant value in devising control measures. Broilers have been recognized as a sensitive chicken species, susceptible to different infections. Coccidiosis is one of the common infections in broiler industry and has been considered to be a management issue rather than protozoan infection round the globe. In the current study, an overall prevalence was recorded as 9.59 %, which is almost comparable to the prevalence reported earlier however, lower than 26.31 %, 46.04 %, 43.89 %, 25.40 %, 39.58 % reported from other parts of the world [14–17]. The range of coccidial infection prevalence has been reported as low as less than 10 % to as high as more than 90 % in broilers globally [18–21]. This lower prevalence recorded in the present study could be due to the following grounds (a) proper litter management (b) proper mixing of anticoccidial in feed (c) timely vaccination (d) environmental conditions. These are the common factors that played a vital role in the spread of coccidiosis in broiler across the world. Coccidiosis is one of the management problem and litter (floor bedding) particulalrly wet litter favours the growth/sporulation of oocyst of coccidia which causes disease in broiler. On the other hand, proper management of litter reduces the chance of getting litter wet, thus minimizing the problem of coccidiosis. In addition, proper mixing of anticoccidial drugs in feed used for preventive measures diminishes chance of disease outbreak. Timely vaccination is very significant in poultry farming as coccidiosis causes immunosuppression [5], so during this immunocompromised phase, the vaccination of birds for other bacterial and viral diseases has less effectiveness. In this study, two Eimeria species were recorded i.e. *E. tenella* and *E. maxima,* which were found to be dorminant in broilers of the studied district [22]. However, various researchers reported these two species along with other *Emeria* species that causes coccidiosis in broiler and other chicken species with varying degree of prevalence [23, 24].. *E. tenella* commonly resides in the caeca of the broiler and considered the most pathogenic [25] comparable to *E. maxima* which is very common and slightly to moderately pathogenic [26], and coccidiosis associated with *E. tenella* is known as caecal coccidiosis (Anonymous et al., 1986). Singh and Meitei [22] reported that higher prevalence of *E.tenella* might be associated with its dominance in nature and high pathogeneicity. Environment factors such as both climate and topography of an area influence the occurence of disease and the main factors that play a vital role in disease outbreak are rainfall, humididty and temperature. Figure 1 explains the pattern of disease prevalence that fluctuates with changing temperature, rain fall and humidity as these parameters are key elements associated with outbreak of coccidiosis and favours the development or sporulation of coccidial oocysts in any particular area [17]. In this study, it has been observed that coccidiosis was present throughout the year with variable monthly prevalence. Season and age [17] also influenced disease occurence. The present study showed that early age group was more prone to the disease [27–30] because of low immune response, development of antibody at early age and higher stocking density [10]. There was statistically significant difference (*P* < 0.05) in prevalence noted between two age groups. In the present study seasonwise highest prevalence was observed in spring season which could be due to favourable environmental parameters for the sporulation of coccidial oocyst as reported elsewhere [30]. However there are other reports of lowest prevalence in summer [31] which confirms findings of the present study. This variation may be due to climate and topography of the area as environmental factors including climate and topography influence disease occurance in any geographical region [3, 19, 32]. Furthermore, better management and extra cares could be the reasons for this difference in seasonal prevalence of coccidiosis in the study area. The difference in prevalence of coccidiosis found was based on floor type of farm as the cemented floor is easy to clean and wash as compared to soiled floor. In soiled floor washing and cleaning are not easy and oocyst of *Eimera* species retainted/trapped in cracks and crevices of soiled floor which when found conducive atmosphere inside the farm starts sporulation thus causing disease in broiler.

## Conclusion

On the basis of results, it was concluded that coccidiosis causing species exist in the area with lower prevalence. Therefore, broiler farmers must be careful about the disease and its causative agents.
